# Recovery Degree of the Natural Flow Regimes and the Corresponding Economic Costs for Reservoir Operation in Fish Spawning Seasons

**DOI:** 10.3390/ijerph16101699

**Published:** 2019-05-14

**Authors:** Cong-Min Liu, Jun Qiu, Fang-Fang Li

**Affiliations:** 1 College of Water Resources & Civil Engineering, China Agricultural University, Beijing 100083, China; S20173091349@cau.edu.cn; 2 State Key Laboratory of Hydroscience & Engineering, Tsinghua University, Beijing 100084, China; aeroengine@tsinghua.edu.cn; 3 State Key Laboratory of Plateau Ecology and Agriculture, Qinghai University, Xining 810016, China

**Keywords:** quantitative relationship, fish spawning, reservoir operation, hydropower generation, upper reaches of the Yellow river

## Abstract

The construction of large-scale reservoirs alters the natural flow process downstream and inevitably affects the aquatic organism. Current studies have verified that flow regimes play an important role in fish spawning stimulus. Recovery of the flow regimes may be incompatible with the economic benefit, mainly referring to hydropower generation. In this study, multiple models are established to study the relationship between the recovery degree of the natural flow regimes and the cost of the hydropower generation in spawning season for different hydrological years. The flow regimes are first quantitatively described by three characteristic parameters including the number of floods, the average duration of each flood, and the daily increment of the natural flow. The model for ecological operation needs to approach these characteristics as close as possible, while the model for economic benefit is set to generate power as much as possible. The ecological flow constraint is also considered to shape the flow process pattern. The proposed methodology is applied on the upper reaches of the Yellow River, where a large-scale reservoir is under planning. Different schemes are compared for different hydrological years to answer the question that to what extent can we recover the flow regime by reservoir operation, and how much the corresponding economic cost is.

## 1. Introduction

The operation of reservoirs directly affects the cyclical fluctuations of the water flow and even leads to the interruption, damaging the healthy river flow formed by natural evolution, destroying the original suitable habitats, and reducing the diversity and biomass of aquatic organisms. Using the established hydrological relationships among flood frequency, flood magnitude, and river-channel capacity, Magilligan et al. [1] develop a scale-independent assessment of the hydrogeomorphic impacts of 21 dams across the United States and found that flow regimes changed profoundly after the construction of the dams. Poff et al. [2] reported that operations of dams tend to homogenize regional flow variability by modifying the magnitude and timing components of high and low flows. Rasanen et al. [3] found that the Mekong’s hydrological regime was significantly altered by the Lancang-Jiang cascade reservoirs, which reduced (increased) the range of hydrological variability during the wet season (dry season). The impoundment of reservoirs has homogenized the flow process, reduced the heterogeneity of habitats and weakened the ecological service function, resulting in reduction of aquatic species. It is necessary to carry out ecological scheduling considering flow regimes to coordinate the power generation and ecological protection downstream [4].

Many studies have considered the relationship between the weighted available area of aquatic habitats or the quality of habitats (indices of fragmentation and connectivity) and water discharge [5,6,7,8,9]. Nevertheless, methods incorporating ecological evaluation or protection can be hardly used in reservoir operation. The difficulty mainly lies in that (1) the ecological benefits is hard to evaluate; (2) the understanding of the ecological effects of flow process is not adequate enough to quantify them. Thus, the ecological needs is evaluated from the perspective of flow, and there are various studies that have identified the ecological flow, in which flow pulses are believed to play a vital role in maintaining the ecological health of rivers [10,11,12]. For example, flow is a major determinant of biotic composition [10,13,14], and it is an effective stimulus for fish spawning, which is at high trophic level in aquatic ecosystems and can be used as indicator species for ecological response to river flow [15,16,17]. Most studies suppose that the natural flow before the disturbance of human activities is the suitable streamflow as the natural flow regime has periodic fluctuations, which is considered to be the main driver of river biodiversity and abundance [5] and an essential factor in maintaining the river environment [18,19,20,21]. However, the natural flow differs in different years, and it is hard to revert it once the dams are constructed. The information of flow regimes implied in the natural flow is more instructive and has more practical significance for reservoir operation. Wang [22] considered that the natural flow regime before hydrological variation is the best ecological flow regime suitable for downstream ecosystems, which can be characterized by a set of parameters. Baron et al. [23] argued that the integrity of freshwater ecosystems depends upon adequate quantity, quality, timing, and temporal variability of water flow. Quantifying the flow regime and extracting characteristic parameters is the prerequisite for optimizing the reservoir release ecologically.

Most of the studies on reservoir operation take a minimum reservoir release as the ecological constraint [14], while in fact, a fixed minimum flow does not result in healthy ecosystems [22]. In addition, such minimum environmental flow is usually determined based on agreement among stakeholders, especially downstream communities. Suen and Eheart [24] believed that the essential anthropocentric approach concentrating on a flat line minimum flow does nothing toward engendering a natural degree of streamflow fluctuation. Yang et al. [25] considered annual variation coefficient and characteristic index of flow when calculating ecological flow. Huang et al. [26] believed that the long-term natural selection makes creatures adapt to the higher frequency of environmental factors, and they improve the Tennant method using the highest frequency monthly flow instead of the monthly mean flow value. Healthy river ecosystems require a natural range of variation in flow [23,27,28], and it is still urgent to consider more complex indicators in ecology flow calculation and find further effective optimization methods. Besides, many studies are based on monthly scale and few studies have not considered the upper limit of ecological flow so that accidental extreme flow events may occur. It is crucial to consider the upper and lower limits of ecological flow especially in daily reservoir operation, which has more practical significances.

As to the operational objectives, most reservoir management strategies optimize hydropower output and producer-economic benefits [29] without considering ecological demand [30,31,32,33,34,35]. Some studies recognized the importance of protecting river ecology and considered ecological flow to be the goal of reservoir optimization. The role flow variability plays in maintaining the ecological health of rivers also begins to be appreciated and incorporated as a goal in reservoir operation [13,36,37,38], and there is general agreement that rivers need to exhibit some resemblance to natural flow variability to support a functional ecosystem. Shiau and Wu [38] evaluated the water flow alterations by parameters of Range of Variability Approach (RVA). After investigating release from Thomson Dam in Victoria, Australia, Harman and Stewardson [10] took a set of flow pulses with particular magnitude, frequency, duration, and set of months as the environmental flow targets. Hughes et al. [12] used natural flow to guide the timing of floods, and developed a heuristic method of reproducing the ‘essential components of flow’—base flows, freshets, and small-medium sized floods by setting monthly low and high flow targets and a target high flow duration. Suen and Eheart [24] used the natural flow regime that is least affected by human activities as the ecological goal, and incorporate flow magnitude, duration, frequency, and timing into flow management strategy to provide the optimal tradeoff between human needs and ecological flow regime maintenance for the Shihmen Reservoir in Taiwan.

Some studies have declared that regulating river flow can improve river ecology without great impact on other benefits [39,40]. Kotchen et al. [41] conducted a benefit-cost analysis of a relicensing agreement for two hydroelectric dams in Michigan and found that the aggregate benefits to society are more than twice as large as the producer costs by changing daily conditions from peaking to run-of-river flows. Babel et al. [42] analyzed the impact of alternative scenarios of a hydropower system operation on energy production and natural flow regime in the La Nga river basin in Vietnam, and concluded that the power production can be increased by 8% and 4% while reducing the overall degree of hydrologic alterations by 24% and 27%, respectively, compared to the existing rule-curve based operation. Wang et al. [43] carried out a two-objective optimization operation study of the Three Gorges Reservoir considering ecological and hydropower benefits, and concluded that the degree of conflict relates to the value of average inflow. The contradiction between hydropower benefits and environmental benefits in dry years is more acute. Taking the Three Gorges-Gezhouba cascade reservoirs as a case study, Dai et al. [44] explored the two-objective optimal dispatching considering the ecological flow shortage index (AEFSI) downstream of the reservoir and hydropower generation. The results showed that the loss of hydropower is very small while optimizing the ecological flow. Research by Zhang et al. [45] showed that mutual restrictions and conflicts between power generation and ecological demand are inevitable, but they can be optimized through multi-objective ecological operation models to fetch the coordinated development of economy and ecology.

This study considers that the flow regime characteristics extracted from natural flow in fish spawning season, which is believed to be beneficial for fish spawning stimulus, represented by the number of floods, duration of each flood, and the daily increment of the flood in spawning season. These three parameters are calculated for wet, normal, and dry years, respectively, before the interference of human activities. Minimizing characteristic difference between the reservoir release and the natural statistics is set as the ecological objective for reservoir operation. On the other hand, maximizing the power generation is conventionally selected as the economic objective. In addition to targeting the optimal ecological flow, the study also considered the basic ecological flow requirements of the river as a constraint for drainage. The daily submaximal and the subminimum flow records in spawning season is used to restrain the daily reservoir release. Genetic Algorithm (GA) is adopted to optimize the models, which are applied on the upper reaches of the Yellow River. Different cases are analyzed to derive the quantitative relationship between ecological effect and hydropower generation in fish spawning seasons for daily reservoir operation.

## 2. Methodology

This study proposes different reservoir optimization models to study the quantitative relationships between hydropower generation and the recovery degree of the natural flow regimes downstream the reservoir in fish spawning seasons in different hydrological years. The models include (A) ecological optimization model with ecological flow constraint, (B) hydropower optimization model with ecological flow constraint, (C) hydropower optimization model without ecological flow constraint, and the optimized operation schemes are compared with the natural flow process without dispatching of reservoir.

### 2.1. Objective Function

#### 2.1.1. Ecological Objective

The ecological objective in this study is to minimize the gap of characteristic parameters between reservoir release and the natural flow process in fish spawning seasons. The natural flow regimes are formed through long-term evolution and can be regarded suitable for aquatic organisms, maintaining the ecological health of rivers. In this study, three characteristic parameters describing the flow process in fish spawning seasons are selected as the indicators of the flow regime, including flood frequency, flood duration, and the change rate of the daily flow. The ecological objective function is mathematically defined as to minimize the gap between the scheduled and natural ecological flow regime, which is defined as the normal value of accumulated hydrological years, as shown in Equation (1); i.e., the closer the characteristic parameters of the optimized flow regime approximates to that of the optimal ecological flow, the less impact of the reservoir operation is on the aquatic ecosystem.

(1) $$Z_{1}=\min\sqrt{{\left(\frac{N-N_{0}}{N_{0}}\right)}^{2}+{\left(\frac{T-T_{0}}{T_{0}}\right)}^{2}+{\left(\frac{\eta -\eta_{0}}{\eta_{0}}\right)}^{2}}$$

where *N*, *T*, and *η* refer to the number of floods (times), average duration of each flood (days), and the daily changing rate ((m^3^/s)/d) of water flow process, respectively. The subscript 0 indicates the normal value of accumulated hydrological years of the natural flow regimes.

#### 2.1.2. Hydropower Objective

Hydropower generation is one of the most important economic purpose of reservoir operation, which may be incompatible with the ecological recovery. To quantify the relationship between the power generation improvement and the recovery degree of the flow regimes, maximizing the power generation is set as another objective function, which is a function of water release and water head, as described in Equation (2):

(2) $$Z_{2}=\max\sum_{i=1}^{n}K\times q_{i}\times \Delta H_{i}$$

(3) $$\Delta H_{i}=L_{i}-T_{i}$$

where *K* is the power output coefficient; *q* is the turbine flow and Δ*H* is the water head, calculated in Equation (3); *L* and *T* are the reservoir water level and the tailwater elevation, respectively; $L_{i}$ is the reservoir water level of day *i*, which is a function of the reservoir storage. $L_{i}$ is varies from day to day with different daily incoming and discharging water, by calculating the daily reservoir storage and according to the storage-elevation curve, $L_{i}$ is obtained by using a piece-wise linearization approach. $T_{i}$ is the tail water level of day *i*, which can be derived from the relationship between turbine release and tail water level. According to the turbine flow, the tail water level of the day *i* is calculated using a piece-wise linearization approach.

### 2.2. Constraints

#### 2.2.1. Water Balance Equation

Reservoir water balance equation is used to calculate the hydrologic connection between adjacent time steps, as described in Equation (4):

(4) $$V_{i}=V_{i-1}+(I_{i}-Q_{i})\times t\;i=1,2,\ldots ,n$$

where *V* is the reservoir storage, *I* is the inflow, *Q* is the reservoir release, and *t* is the discharge time of each period.

#### 2.2.2. Water Level Constraint

The water level in the reservoir needs to be in a certain range to prepare for the unexpected flood and also avoid overtopping, as shown in Equation (5):

*L_min_* ≤ *L_i_* ≤ *L_max_i* = 1, 2, …, *n* (5)

where *L_min_* and *L_max_* are the regulation-defined minimum and maximum water level, respectively.

#### 2.2.3. Hydropower Output Constraint

The hydropower output $P_{i}$ needs to guarantee the firm power $P_{min}$ and also be within the installed capacity of the hydropower station *P_max_*, as illustrated in Equation (6)

(6) $$P_{min}\leq P_{i}\leq P_{max}\;i=1,2,\ldots ,\;n$$

#### 2.2.4. Turbine Working Constraint

The hydropower units need to work under a certain hydrological condition, and the turbine flow should be limited within a certain range, as shown in Equations (7) and (8):

(7) $$h_{min}\leq h_{i}\leq h_{max}$$

(8) $$q_{min}\leq q_{i}\leq q_{max}$$

where $h_{min}$ and $h_{max}$ are the minimum and maximum water head, respectively, required for the normal operation of the turbine; $q_{min}$ and $q_{max}$ are the minimum and maximum turbine flow respectively required for the normal operation of the turbine.

#### 2.2.5. Ecological Flow Constraint

The ecological flow constraint refers to the reservoir release, which needs to be within a certain range according to the historical records of the natural flow processes. The determination of the boundary of the flow process is based on the undisturbed natural flow records. In this study, the historical years are firstly classified into dry years, normal years, and wet years. The subminimal and submaximal daily flow of different years in fish spawning season are set as the ecological flow boundaries, as show in Equation (9). The reason why not choose the maximum and minimum values directly is that there exist occasional abnormal flow values that are unfavorable to the river habitat in the natural flow process, and the secondary maximum and minimum flow processes can ensure that the flow does not threaten the river habitat to a greater extent. The daily ecological flow constraints are different from day to day, and thus, the optimized results have the variation of the natural flow regime.

Taking the reservoir release as the decision variables, such ecological constraint also changes the initial solution space and thus improves the efficiency of the optimization algorithms.

(9) $$Q_{i}^{2^{nd}\_min}\leq Q_{i}\leq Q_{i}^{2^{nd}\_max}\;i=1,2,\ldots ,n$$

### 2.3. Optimization Models

In this study, three models A–C listed in Table 1 are optimized, respectively, to explore the tradeoff between hydropower generation and the recovery degree of the natural flow regime, which are also compared with the natural case D.

The detailed models are illustrated as below.

A: *Minimize Z*_1_ in Equation (1) *s.t.* Water balance constraint Equation (4) Water level constraint Equation (5) Hydropower constraint Equation (6) Turbine working constraint Equations (7) and (8) Ecological flow constraint Equation (9)
B: *Maximize Z*_2_ in Equation (2) *s.t.* Water balance constraint Equation (4) Water level constraint Equation (5) Hydropower constraint Equation (6) Turbine working constraint Equations (7) and (8) Ecological flow constraint Equation (9)
C: *Maximize Z*_2_ in Equation (2) *s.t.* Water balance constraint Equation (4) Water level constraint Equation (5) Hydropower constraint Equation (6) Turbine working constraint Equations (7) and (8)
D: Natural flow regime (no optimization) Calculate ecological objective in Equation (1) Calculate hydropower objective in Equation (2) *s.t.* Hydropower constraint Equation (6) Turbine working constraint Equations (7) and (8)

### 2.4. Implementation

For each of the models A, B, and C, Genetic Algorithm (GA) is selected as the optimization algorithm, and the results are compared to analyze the quantitative relationship between the power generation and the recovery degree of flow regime. Since both of the objective values in Equations (1) and (2) are determined by the reservoir discharge *Q*, it is set to be the decision variable, as shown in Equation (10). Firstly, a population of candidate operation schemes in Equation (10) are generated randomly in the solution space, and the population size in this study is set to be 100. Each of the individual in the population is evaluated by the objective function in Equations (1) or (2), also known as the fitness of the individual. Those individuals with better fitness have larger chance to survive and to evolve with crossover and mutation to generate a new generation of individuals, which are then evaluated again to preserve those ones with better fitness. All the constraints from Equations (5) to (9) are either used to judge whether the individual is feasible or considered with penalty functions.

(10) $$\vec{Q}=Q_{1},Q_{2},\ldots ,Q_{n}$$

## 3. Case Study

### 3.1. Study Area

The Yellow River is the second longest river in China with the length of 5464 km and the drainage area of 752,400 km^2^. The upper and middle reaches of the Yellow River account for 97% of the total area of the basin. The western part of the basin belongs to the Qinghai-Tibet Plateau, with an altitude of over 3000 m.

Currently, the Longyangxia Reservoir is the first large-scale reservoir on the upper reaches of the Yellow River, while other large-scale Yangqu reservoirs are planned upstream. The Yangqu Hydropower Station is mainly composed of dam, left flood discharge building, and right diversion power generation building. The dam crest elevation is 2721.0 m and the maximum height is 150 m. The normal storage water level of Yangqu reservoir is 2715.0 m, the corresponding storage capacity is 1472 × 10^6^ m^3^, the dead water level is 2713.0 m, and the regulating storage capacity is 95.6 × 10^6^ m^3^. The installed capacity of the power station is 1200 MW. There are no comprehensive utilization requirements for irrigation, water supply, flood control, and shipping between Yangqu and Longyangxia. The downstream water supply and flood control tasks of Longyangxia are undertaken by Longyangxia Reservoir. The main task of Yangqu Hydropower Station development is power generation [46].

**Figure 1:**
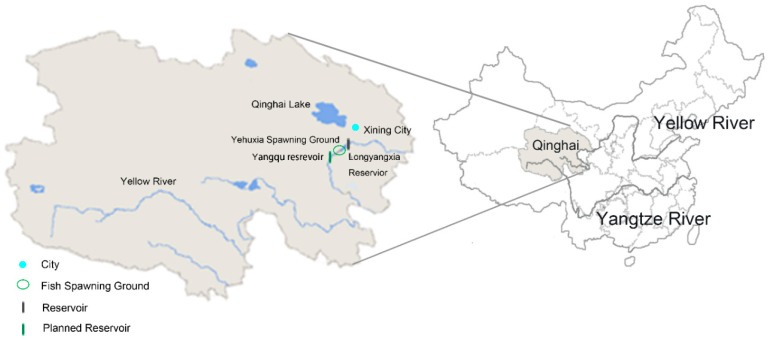
Study area.

An important issue to demonstrate before the reservoir construction is to what extent the reservoir will affect the ecosystem, and to what extent the natural flow regimes downstream can be recovered by reservoir operation. Since the area is untraversed, the main ecological issue on the upper reaches of the Yellow River is the impact on the fish, especially in the fish spawning season, when the fishes need a certain flow regime to stimulate spawning. Thus, the flow regime at an important fish spawning ground called Yehuxia, about 80 km upstream the Longyangxia Reservoir, is studied, as indicated in Figure 1.

There are several rare indigenous fish distributed in this section, mainly including Gymnocypris eckloni Herz, Platypharodon extremus Herz, Gymnodiptychusd. Pachycheilus Herz, Schizopygopsis phlzovi Kessler, Triplophysa pseudoscleroptera, Trilophysa brevviuda, Cobitis sinensis Sauvage et Dabfy, Triplophysa stenura, Triplophysa siluroides, Triplophysa pappenheimi, and Triplophysa leptosome [47]. Most of these plateau cold water fish lay eggs in April to June, and thus, these three months are defined as the spawning season in this case.

### 3.2. Analysis of Natural Flow Regimes

**Figure 2:**
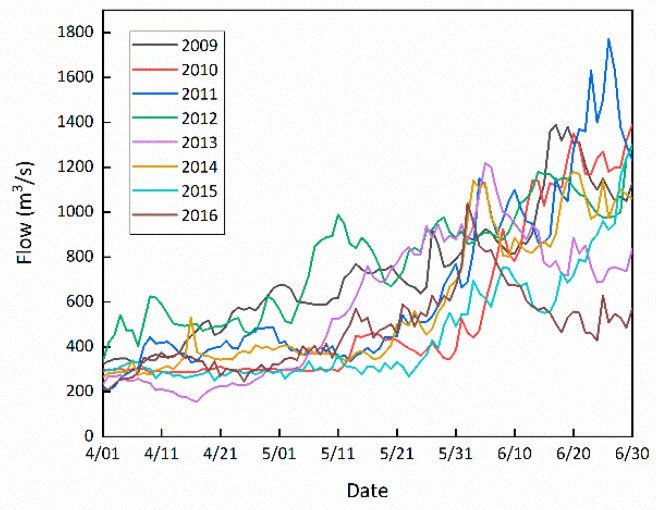
Natural flow processes in different years.

Figure 2 shows the natural flow process at the Yehuxia spawning ground during the spawning season. The difference of the flow regimes in different years are quite large, especially in water volume. It is necessary to classify the wet years, normal years, and dry years according to the water volume.

The Pearson III curve is used to fit the annual average flow over the years. According to the national “Hydrological Basic Terminology and Symbol Standards” (GB/T50095-98), the natural flow sequence from 2009 to 2016 is classified into different hydrological years, as shown in Table 2. According to the principle of high water volume and unfavorable distribution, i.e., the amount of water in the flood season is relatively high, the year of 2011, 2014, and 2015 are selected as the representative year for wet years, normal years, and dry years, respectively, and studied.

The characteristic parameters of the natural flow regimes in the spawning season of different years including the number of floods (times), duration (days), and the daily average increase ((m^3^/s)/d) are shown in Table 2, as well as the recommended average values for different hydrological years. Thus, the gap between the natural and scheduled flow regimes can be analyzed to quantify the recovery degree of the ecosystem.

### 3.3. Ecological Flow Constraint Based on Historical Flow Process

**Figure 3:**
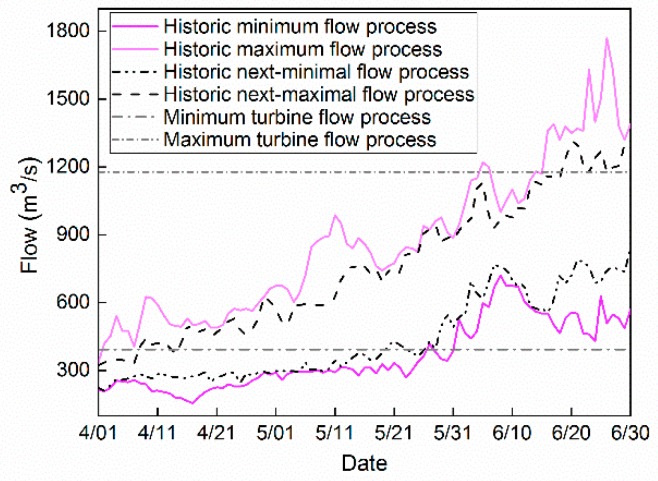
Schematic diagram of the ecological flow constraint.

According to the daily flow sequence at the Yehuxia spawning ground in different hydrological years, the daily maximum and minimum flow processes, as well as the daily secondary maximum and secondary minimum flow processes are calculated, as shown in Figure 3. It can be seen that there are some occasional abnormal flow values with the maximal and minimal values that should be unfavorable to the river habitat, and thus, the secondary maximum and secondary minimum flow processes are set as the suitable ecological flow boundaries, in which the candidate reservoir release schemes are generated to be optimized.

The two horizontal lines in Figure 3 are the maximum and minimum flow allowed by the normal operation of the turbine. When the flow is smaller than the minimum turbine flow, the turbine cannot generate hydropower efficiently, while if the flow is larger than the maximum turbine flow, some of the water needs to be abandoned from the spillway.

## 4. Results

**Figure 4:**
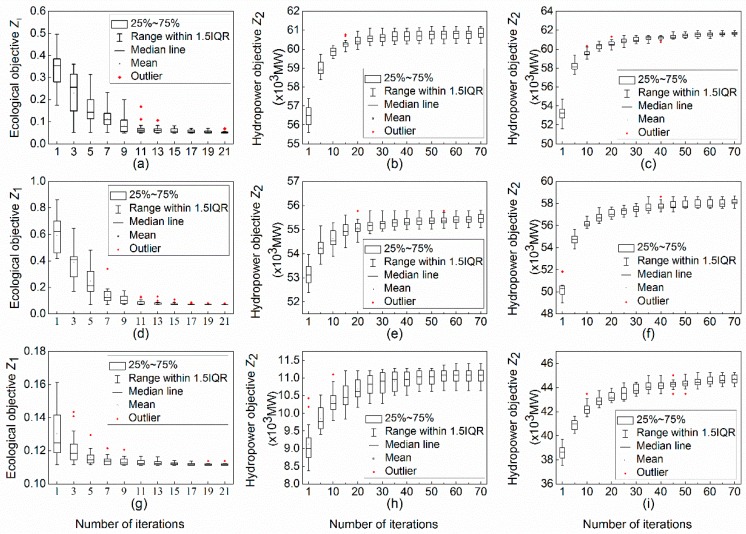
Convergence procedures of different models with 20 trials in different hydrological representative years, where (**a**–**c**) stands for model A, model B, and model C in the wet year (2011), respectively; (**d**–**f**) stands for model A, model B, and model C in the normal year (2014), respectively; (**g**–**i**) stands for model A, model B, and model C in the dry year (2015), respectively.

Since GA is a metaheuristic algorithm, starting with a group of random seeds, it is necessary to check the stability of the algorithm with multiple trials. Figure 4 shows the convergence procedures of different models for different hydrological representative years, as well as the distribution of the solutions in the procedure for different trials.

It can be seen from Figure 4 that for model A, i.e., (a), (d), and (g), that the optimizations basically converge within 25 iterations even for different trials. Due to the limitation of natural inflow process, there exists a gap between the regimes of reservoir release and the optimum flow thus the objective function Z_1_ is not 0 when it is convergent. As to Figure 4b,c,e,f,h,i, it needs about 70 iterations for model B and model C to be convergent. In addition, the size of the boxes indicates that model A is more stable than model B and model C.

Table 3 shows the optimal objective values with model A, B, and C, as well as the simulative results of the model D; Table 4 shows the optimization ratio of the objectives compared with the natural flow; and Table 5 shows the relationship between the recovery degree represented by the ecological objective and the economic benefit represented by the hydropower objective for different models.

For model D, reservoir releases as much water as the natural inflow, and only the objective value in Equations (1) and (2) are calculated.

Model C aims to maximize the hydropower generation without consideration of the impact of the changes of the flow regime. In the wet year of 2011, normal year of 2014, and dry year of 2015, the hydropower objective values for model C are the largest among different models, which is increased by 18.71%, 33.58%, and 61.46%, respectively, compared with the natural flow, while the ecological objective is decreased by 4999.90%, 2051.10%, and 1303.11%, respectively.

Model B considers the ecological flow constraint when maximizing hydropower generation. Ecological flow restriction refers to limiting the daily reservoir discharge within the historical secondary-maximum and secondary-minimum flow values. Compared with the natural flow process, the hydropower objectives in the wet year of 2011 and normal year of 2014 are improved by 16.29% and 26.98%, respectively, and the dry year of 2015 is decreased by 60.14%, which are obviously less than model C. Its ecological objective is decreased by 528.63%, 329.08%, and 17.23%, respectively. Compared with model C, the ecological impact is alleviated.

Model A is committed to fully optimizing the river flow process with the goal of recovering the natural flow process, and also considers ecological flow constraint. Compared with natural state in model D, in wet year of 2011, normal year of 2014, and dry year of 2015, the ecological objectives are optimized by 50.98%, 69.54%, and 67.00%, respectively. The reason that the ecological value is even better than the natural state is that the optimization objective is the multiple year average value, and the natural situation of a specific year may be worse than the standard. And its hydropower objective improved by 7.37% and 16.10% in the wet year of 2011 and normal year of 2014, respectively, and decreased by 75.42% in the dry year of 2015.

Table 4 and Table 5 show that model A restricts the flow process and consequently protects the river ecological environment.

**Figure 5:**
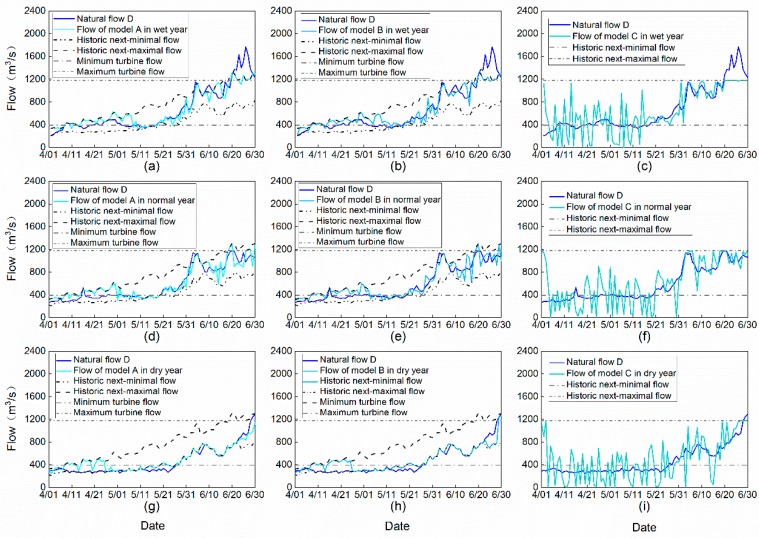
Optimum flow processes corresponding to different models in different hydrological representative years, where (**a**–**c**) stands for model A, model B, and model C in the wet year (2011), respectively; (**d**–**f**) stands for model A, model B, and model C in the normal year (2014), respectively; (**g**–**i**) stands for model A, model B, and model C in the dry year (2015), respectively.

Figure 5 shows the flow processes corresponding to the optimum with different models for the representative hydrological years.

**Figure 6:**
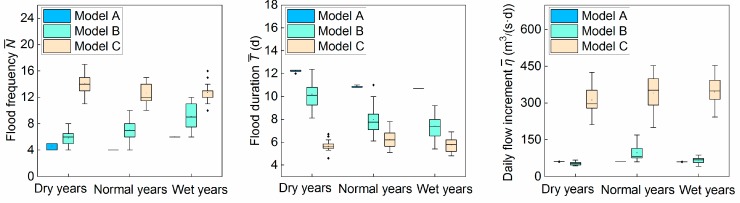
Box diagram of characteristic parameters of flow rising process in 20 operational results.

Figure 6 shows the box diagrams of the characteristic parameters of flow rising processes resulting from each model in 20 trials. It can be seen that the boxes of the three parameters from model A in different hydrological years are relatively flat, i.e., the results of 20 trials are stable and almost tend to the same value. Model B and C result in lanky boxes, and there even appear to be abnormal values with large deviation, which indicate that the results of 20 trials are unstable.

Table 6 shows the comparison of the characteristics of the flow rising process derived from different models, i.e., the number of floods, the average duration of floods, and the daily increment of the flood.

## 5. Discussion

It is clear that there, indeed, exists some competitive relationship of the water between power generation and ecological recovery. However, Table 4 and Table 5 indicate that recovering the flow regimes only costs around 10% of the power generation in fish spawning season in wet and normal years, i.e., the ecological recovery does not cost the power generation as great as expected.

Figure 5 shows that flow process corresponding to model A and model B is within the ecological constraints. When the inflow exceeds the ecological scope, the operation of the reservoir regulates the flow process and consequently protects the downstream ecological environment. However, their optimization objectives are different, which can be further analyzed by flow parameters. Obviously, the flow process of model C is not within the ecological range. The flow fluctuates greatly near the water inflow process with large amplitude, which is not conducive to the aquatic ecosystem downstream.

Table 6 indicates that the characteristics of the flow derived from model C has the biggest gap with the natural state in all the wet, normal, and dry years, especially the daily average increment of the flood. Compared with model C, the parameters from model B are improved, but still differ from the parameters of the optimal flow regime. The three parameters of the flow process of model A are basically consistent with the standard, and it is even better than those of the representative year derived from model D, which is more suitable for spawning and reproduction of aquatic organisms.

Since the hydropower, and ecological objectives seem to reflect objectives which are conflicting in nature in this study, the tradeoff between the two objectives could be further studied using Pareto solutions.

## 6. Conclusions

A certain flow regime is in need in fish spawning season to stimulate spawning, which will be altered by reservoir operation. In this study, the recovery degree of the natural flow regimes and the corresponding economic costs for reservoir operation is studied in fish spawning seasons. Three characteristic parameters including the number of floods, the average duration of the floods, and the daily average increment of the flood are used to describe a flow regime. The historical flow data of different hydrological years are used to determine the preferred parameters. Different models with the ecological and hydropower objective are established to study the relationship of ecological recovery degree and the power generation. The proposed models are applied to the upper reaches of the Yellow River. The result verifies the competitive relationship between ecological recovery and power generation, and more importantly, it indicates that the river flow regime can be significantly recovered by proper scheduling at the cost of around 10% of the hydropower generation in wet and normal years.

## Figures and Tables

**Table 1:** Comparison for different models.

Model	Ecological Objective in Equation (1)	Hydropower Objective in Equation (2)	Ecological Flow Constraint in Equation (9)
A	√	×	√
B	×	√	√
C	×	√	×
D	Calculated	Calculated	×

Note: √ means the corresponding Equation is considered; and × means it has been omitted.

**Table 2:** Characteristic parameters of natural flow process in different years.

	Year	N (times)	$\bar{N}$ (times)	$\bar{T^{\prime }}$ (d)	$\bar{T}$ (d)	$\bar{\eta^{\prime }}$ ((m^3^/s)/d)	$\bar{\eta }$ ((m^3^/s)/d)
Wet years	2009	6	6.3	11.3	10.7	50.7	59.4
	**2011**	6		10.5		64.3	
	2012	7		10.4		63.3	
Normal years	2010	5	4.3	8.2	10.9	65.7	61.7
	2013	4		12.8		70.2	
	**2014**	4		11.8		49.1	
Dry years	**2015**	3	4.5	12.7	12.2	58.5	60.4
	2016	6		11.8		62.2	
Average	-	-	5.0	-	11.3	-	60.5

Note: The black fonts indicate the representative years.

**Table 3:** Optimal objective values with different models.

	Model	Wet Year 2011	Normal Year 2014	Dry Year 2015
Ecological objective *Z*_1_	A	0.0476	0.0704	0.1113
	B	0.6104	0.9916	0.3954
	C	4.9520	4.9712	4.7327
	D	0.0971	0.2311	0.3373
Hydropower objective *Z*_2_ (×10^3^ MW)	A	56.36	50.99	6.92
	B	61.04	55.77	11.22
	C	62.31	58.67	45.45
	D	52.49	43.92	28.15

**Table 4:** Optimization ratio of the objectives compared with the natural flow.

		Wet Year 2011	Normal Year 2014	Dry Year 2015
Ecological objective	($Z_{1}^{D}$ − $Z_{1}^{A}$)/$Z_{1}^{D}$	50.98%	69.54%	67.00%
	($Z_{1}^{D}$ − $Z_{1}^{B}$)/$Z_{1}^{D}$	−528.63%	−329.08%	−17.23%
	($Z_{1}^{D}$ − $Z_{1}^{C}$)/$Z_{1}^{D}$	−4999.90%	−2051.10%	−1303.11%
Hydropower objective	($Z_{2}^{A}$ − $Z_{2}^{D}$)/$Z_{2}^{D}$	7.37%	16.10%	−75.42%
	($Z_{2}^{B}$ − $Z_{2}^{D}$)/$Z_{2}^{D}$	16.29%	26.98%	−60.14%
	($Z_{2}^{C}$ − $Z_{2}^{D}$)/$Z_{2}^{D}$	18.71%	33.58%	61.46%

**Table 5:** Relationship between the recovery degree represented by ecological objective and the economic benefit represented by hydropower objective for different models.

		Wet Year 2011	Normal Year 2014	Dry Year 2015
Ecological objective	($Z_{1}^{C}$ − $Z_{1}^{A}$)/$Z_{1}^{C}$	99.04%	98.58%	97.65%
	($Z_{1}^{B}$ − $Z_{1}^{A}$)/$Z_{1}^{B}$	92.20%	92.90%	71.85%
	($Z_{1}^{C}$ − $Z_{1}^{B}$)/$Z_{1}^{C}$	87.67%	80.05%	91.65%
Hydropower objective	($Z_{2}^{A}$ − $Z_{2}^{C}$)/$Z_{2}^{C}$	−9.55%	−13.09%	−84.77%
	($Z_{2}^{A}$ − $Z_{2}^{B}$)/$Z_{2}^{B}$	−7.67%	−8.57%	−38.32%
	($Z_{2}^{B}$ − $Z_{2}^{C}$)/$Z_{2}^{C}$	−2.04%	−4.94%	−75.31%

**Table 6:** Comparison of three ecological parameters.

	$\bar{N}$	$\bar{T}$	$\bar{\eta }$	Ecological Objective *Z*_1_
**Wet years**	**6.3**	**10.7**	**59.4**	**0**
A	6.0	10.70	59.42	0.0476
B	9.1	7.41	66.61	0.6104
C	12.7	5.75	345.33	4.9520
D	6.0	10.50	64.30	0.0971
**Normal years**	**4.3**	**10.9**	**61.7**	**0**
A	4.0	10.85	61.70	0.0704
B	7.05	7.98	96.30	0.9916
C	12.65	6.24	340.79	4.9712
D	4.0	11.80	49.10	0.2311
**Dry years**	**4.5**	**12.2**	**60.4**	**0**
A	4.6	12.23	60.39	0.1113
B	5.80	10.19	52.88	0.3954
C	14.05	5.65	312.27	4.7327
D	3.0	12.70	58.50	0.3373

Note: The bold font in the tale indicates the statistical standard from historical records.
